# Pyrrolo[3,2-*c*]pyridine derivatives with potential inhibitory effect against FMS kinase: *in vitro* biological studies

**DOI:** 10.1080/14756366.2018.1491563

**Published:** 2018-08-02

**Authors:** Mohammed I. El-Gamal, Chang-Hyun Oh

**Affiliations:** a Department of Medicinal Chemistry, College of Pharmacy, University of Sharjah, Sharjah, United Arab Emirates;; b Sharjah Institute for Medical Research, University of Sharjah, Sharjah, United Arab Emirates;; c Department of Medicinal Chemistry, Faculty of Pharmacy, University of Mansoura, Mansoura, Egypt;; d Center for Biomaterials, Korea Institute of Science and Technology, Seoul, Republic of Korea;; e Department of Biomolecular Science, University of Science and Technology, Daejeon, Republic of Korea

**Keywords:** CSF-1R, FMS, kinase inhibition, pyrrolo[3,2-c]pyridine

## Abstract

A series of eighteen pyrrolo[3,2-*c*]pyridine derivatives were tested for inhibitory effect against FMS kinase. Compounds **1e** and **1r** were the most potent among all the other tested analogues (IC_50_ = 60 nM and 30 nM, respectively). They were 1.6 and 3.2 times, respectively, more potent than our lead compound, **KIST101029** (IC_50_ = 96 nM). Compound **1r** was tested over a panel of 40 kinases including FMS, and exerted selectivity against FMS kinase. It was further tested against bone marrow-derived macrophages (BMDM) and its IC_50_ was 84 nM (2.32-fold more potent than **KIST101029** (IC_50_ = 195 nM)). Compound **1r** was also tested for antiproliferative activity against a panel of six ovarian, two prostate, and five breast cancer cell lines, and its IC_50_ values ranged from 0.15–1.78 µM. It possesses also the merit of selectivity towards cancer cells than normal fibroblasts.

## Introduction

Colony-stimulating factor-1 receptor (CSF-1R), or FMS, kinase is a member of type III receptor tyrosine kinases family. It interacts with CSF-1 or Il-34, and the produced signal transduction results in proliferation and survival of the monocyte/macrophage lineage cells^1^^,^^2^. FMS kinase is over-expressed in different cancer types (e.g. ovarian^3^, prostate^4^, and breast^5–7^) as well as inflammatory disorders (e.g. rheumatoid arthritis)^8^. So FMS inhibitors could be useful drug candidates for treatment of these disorders.

**Figure 1.**
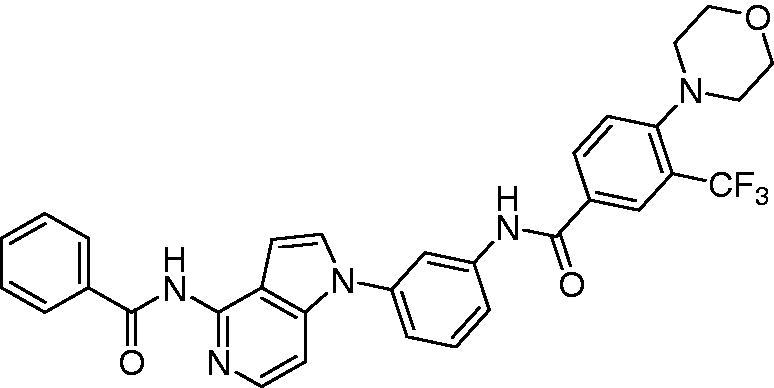
Structure of the lead compound, **KIST101029**.

Several FMS kinase inhibitors of different chemical classes have been recently reviewed by our group^9^. Among them, diarylamide derivatives have been reported^10–15^. We have previously reported a diarylamide derivative (**KIST101029**, Figure 1) as a potent and selective FMS kinase inhibitor^16^. It exerted also high potency against ovarian, prostate, and breast cancer cell lines^17^.

In the present study, we tested a series of diarylamides and diarylureas possessing pyrrolo[3,2-*c*]pyridine scaffold against FMS kinase. Many of them were previously reported as antiproliferative agents^18–20^. The most potent compound against FMS kinase was tested against a panel of 40 kinases including FMS, tested against a panel of 13 cancer cell lines of 3 different cancer types and HS 27 fibroblasts, and against bone marrow-derived macrophages (BMDM) in order to investigate its biological effects. The results were compared with those of the lead compound **KIST101029**. The results, structure-activity relationship (SAR) studies, and relevant discussions are presented in details.

## Experimental

### Synthesis of the target molecules 1a–q

Compounds **1f**, **1m–o**, and **1q** are new, while the other 12 compounds are previously reported^18–20^. In addition, compound **1r** is also new. The synthetic procedure utilised for its synthesis as well as its spectral data are reported in the next section. The synthetic procedures, purification methods, and the spectral analysis data for compounds **1a–q** have been provided in the supplementary file.

### Synthesis of compound 1r

A mixture of compound **8b** (24 mg, 0.1 mmol), 4-morpholino-3-(trifluoromethyl)benzoic acid (57 mg, 0.2 mmol), HOBt (31 mg, 0.22 mmol), and EDCI (50 mg, 0.26 mmol) in dry DMF (2.0 ml) was cooled to 0 °C in inert atmosphere. TEA (0.04 ml, 0.02 mmol) was added thereto at the same temperature. The mixture was then stirred at 80 °C for 12 h. The reaction mixture was cooled and then partitioned between water (10 ml) and ethyl acetate (15 ml), and the organic layer was separated. The aqueous layer was then extracted with ethyl acetate (2 × 10 ml) and the combined organic phase was washed with brine and dried over anhydrous sodium sulphate. The organic solvent was evaporated, and the residue was purified by column chromatography (silica gel, hexane-ethyl acetate 4:1 v/v followed by 1:1 v/v) to get the pure compound. Yield 7.5%; ^1^H NMR (400 MHz, CDCl_3_): *δ* 8.56 (s, 1H), 8.39 (s, 1H), 8.17–8.07 (m, 2H), 7.71–7.53 (m, 2H), 7.39 (brd, 2H, *J* = 8.6 Hz), 7.34 (t, 1H, *J* = 3.2 Hz), 7.30 (brd, 1H, *J* = 8.0 Hz), 7.13 (brs, 2H), 6.83 (brd, 1H, *J* = 7.7 Hz), 6.74 (dd, 1H, *J* = 1.9 Hz and 1.5 Hz), 3.86 (t, 4H, *J* = 2.6 Hz), 3.02 (t, 4H, *J* = 2.1 Hz); LC-MS: 482.1 (M^+^ +1), 481.1 (M^+^); elemental analysis: Calculated: C: 62.36%, H: 4.61%, N: 14.55%, Found: C: 62.23%, H: 4.50%, N: 14.68%.

### Bioactivity protocol

#### In vitro kinase screening

Reaction Biology Corp. Kinase HotSpot^SM^ service [http://www.reactionbiology.com] was utilised for screening of the target compounds as per the protocol reported in the literature^21^. In order to calculate the IC_50_ values and the inhibition percentages at different concentrations, each compound was tested in 10-dose duplicate assay mode starting with 81 µM and threefold serial dilution.

#### Antiproliferative screening against cancer cell lines

Compounds **1r** and **KIST101029** were tested at the National Cancer Institute (NCI, Bethesda, MD) following their standard protocol [https://dtp.cancer.gov/discovery_development/nci-60/methodology.htm].

#### Antiproliferative screening against HS 27 fibroblasts

It was conducted following the previously reported protocol^17^.

#### BMDM assay

The macrophages were isolated from C57BL6 murine bone marrow using Miltenyi Biotec MACS beads (CD11b). The macrophages (30,000 cells/well) were cultured in RPMI 1640 medium containing 10% foetal bovine serum, 2-mercaptoethanol (50 mM), and the test compound for 1 h at 37 °C in the presence of 5% CO_2_. CSF-1 (20 ng/mL) was then added and the system was incubated for 5 days. Each compound was tested in a 10-dose testing mode, threefold serial dilution starting with 1 µM concentration. The inhibitory effects of the tested compounds against CSF-1-induced BMDM growth were determined using Alamar Blue^TM^ (Serotec).

## Results and discussion

### Chemistry

**Scheme 1.**
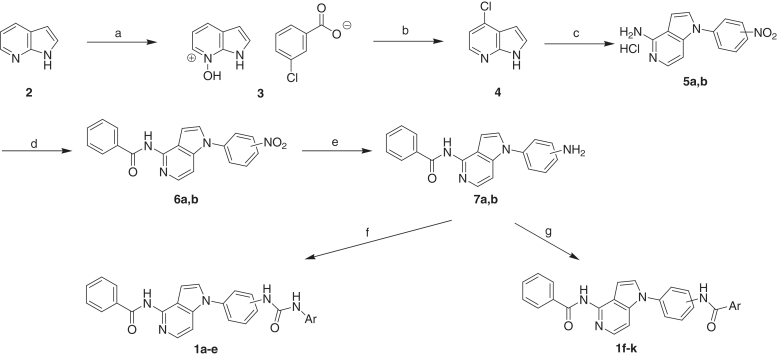
Reagents and conditions: (a) 3-chloroperoxybenzoic acid, DME:heptane (1:2), rt, 2.5 h; (b) POCl_3_, 55 °C then rt then 85–90 °C, 18 h; (c) appropriate nitroaniline, 180 °C, 2–5 h; (d) benzoyl chloride, diisopropylamine, CH_3_CN, rt, 8 h; (e) Pd/C, H_2_, THF, rt, 2 h; (f) aryl isocyanate, THF, rt, 8 h; (g) benzoic acid derivative, HOBt, EDCI, TEA, DMF, 80 °C, 12 h.

The 4-benzamidopyrrolo[3,2-*c*]pyridine derivatives **1a–k** were synthesised as per the pathway illustrated in Scheme 1. Pyrrolo[2,3-*b*]pyridine (**2**) was treated with *m*-chloroperoxybenzoic acid to produce the *m*-chlorobenzoate salt **3**. Heating compound **3** with phosphorus oxychloride yielded 4-chloropyrrolo[2,3-*b*]pyridine (**4**)^22^. Fusion of compound **4** with the appropriate nitroaniline yielded 1-aryl-4-aminopyrrolo[3,2-*c*]pyridine HCl salts (**5a,b**) after ring rearrangement^23^. The amines **5a,b** were treated with benzoyl chloride in the presence of diisopropylamine to produce the corresponding benzamido analogues **6a,b**. The nitro group of compounds **6a,b** was reduced using hydrogen gas and Pd/C to yield the corresponding aniline derivatives **7a,b**. In order to get the diarylurea products **1a–e**, compounds **7a,b** were reacted with appropriate aryl isocyanate at room temperature. On the other hand, heating the amines **7a,b** with the appropriate benzoic acid derivative in the presence of EDCI, HOBt, and triethylamine led to condensation reaction and formation of the bisamide products **1f–k**^18–20^.

**Scheme 2.**
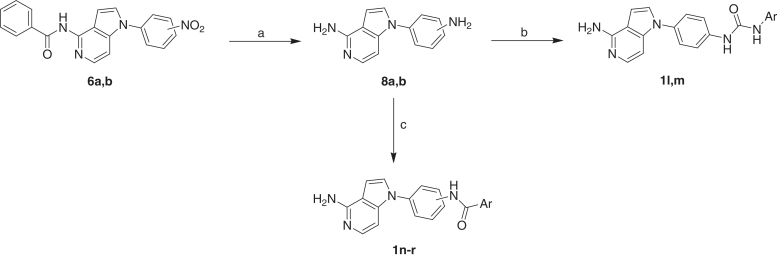
Reagents and conditions: (a) SnCl_2_.H_2_O, EtOH, reflux, 4 h; (b) aryl isocyanate, THF, rt, overnight; (c) benzoic acid derivative, HOBt, EDCI, TEA, DMF, 80 °C, 12 h.

Reaction of compounds **6a,b** using stannous chloride refluxing ethanol led to reduction of the nitro group and hydrolysis of the benzamido moiety to yield the diamine derivatives **8a,b**. The aniline amino group is more reactive in the next reactions than the other amino attached to the heterocyclic nucleus due to higher electron density and higher nucleophilicity. Formation of the urea products **1l,m** and the amide derivatives **1n–r** (Scheme 2) was carried out by the same methods described for synthesis of compounds **1a–e** and **1f–k**, respectively (Scheme 1).

### Biology

#### Kinase screening results and discussion

Compounds **1a–r** were tested in a 10-dose testing mode starting with 81 µM, threefold serial dilutions, against FMS kinase in a cell-free enzyme assay. The IC_50_ values are summarised in Table 1. Upon comparing the structures and potencies, it was found that compounds **1c**, **1e**, and **1g** possessing benzamido moiety at position 4 of the pyrrolopyridine nucleus were more potent than the corresponding primary amino analogues **1 l**, **1m**, and **1o**. This can be due to occupancy of a hydrophobic pocket with that additional benzoyl moiety and/or formation of additional hydrogen bond by the carbonyl oxygen. On the contrary, the amino analogues **1p** and **1r** were more potent than the corresponding benzamido derivatives **1j** and **KIST101029**.

**Table 1.** Structures of the target compounds and their IC_50_ values against FMS kinase. 
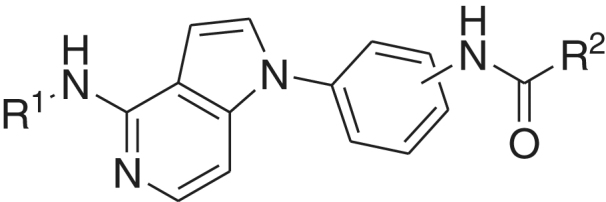

Compound No.	R1	Site of attachment to benzene ring	R2	IC50 (μM)^a^
**1a**	Bz	*para*		0.19 ± 0.02
**1b**	Bz	*meta*		1.43 ± 0.29
**1c**	Bz	*para*		0.39 ± 0.02
**1d**	Bz	*meta*		2.47 ± 0.03
**1e**	Bz	*para*		**0.06 ± 0.01**
**1f**	Bz	*para*		>81
**1g**	Bz	*meta*		1.27 ± 0.07
**1h**	Bz	*para*		10.80 ± 0.60
**1i**	Bz	*meta*		>81
**1j**	Bz	*para*		33.15 ± 0.45
**1k**	Bz	*para*		>81
**1l**	H	*para*		3.20 ± 0.07
**1m**	H	*para*		1.45 ± 0.03
**1n**	H	*para*		>81
**1o**	H	*meta*		40.70 ± 2.40
**1p**	H	*para*		0.10 ± 0.03
**1q**	H	*para*		>81
**1r**	H	*meta*		0.03 ± 0.02
**KIST101029**				0.096

^a^IC_50_ values are expressed as means of 10-dose duplicate assay ± SEM.

The central phenyl ring attached to *N*1 atom of the pyrrolopyridine nucleus was *para*-disubstituted in some derivatives or *meta*-disubstituted in others. The results showed that *para*-disubstituted compounds **1a**, **1c**, and **1h** were more potent than the corresponding *meta*-disubstituted derivatives **1b**, **1d**, and **1i**. On the other hand, the *meta*-disubstituted compounds **1g**, **1o**, and **1r** showed stronger inhibitory effect against FMS kinase than the *para*-disubstituted positional isomers **1f**, **1n**, and **1p**. These differences in the substitution position can affect proper orientation and fitting at the active site.

The urea derivatives **1a**, **1b**, **1e**, and **1m** were generally more potent than the corresponding analogues with amide linker **1h**, **1i**, **1k**, and **1q**. The urea linker is longer and can be optimal for best fitting at the binding site. The urea spacer contains also additional *NH*, compared with the amide. This terminal *NH* could contribute to stronger affinity by additional hydrogen bonding. Any or both of these effects can account for stronger enzyme inhibition effect of the urea derivatives.

Upon investigating the terminal aryl ring effect on the activity, it was found that 3′,5′-bis(trifluoromethyl)phenyl and 4′-morpholino-3′-(trifluoromethyl)phenyl are the best rings in diarylureas and diarylamides, respectively. These rings may contribute to stronger affinity with the enzyme. The morpholino moiety is also a polar ring, which enhances the solvent exposure and aqueous solubility of the molecule.

Among all the tested compounds, **1e** and **1r** were more potent than the lead compound **KIST101029**. Their potencies were 1.6 and 3.2 times, respectively, higher than that of **KIST101029**. Compound **1r** possessing primary amino, *meta*-disubstituted central phenyl, amide linker, and 4′-morpholino-3′-(trifluoromethyl)phenyl terminal ring was the most potent among this series of compounds. So we decided to consider it for further biological investigations.

Compound **1r** was tested against a panel of 40 kinases including FMS. The inhibition percentages at 1 µM concentration are summarised in Table 2. It showed selectivity against FMS kinase with 81% inhibition. Its inhibition percentages against FLT3 (D835Y) and c-MET kinases were 42% and 40%, respectively. So its IC_50_ values against both of them will be in micromolar scale. Compared with its IC_50_ values against FMS kinase (30 nM, Table 1), it can be concluded that compound **1r** is more than 33 times more selective towards FMS than the other kinases. The inhibition percentages exerted by compound **1r** against the other 37 tested kinases were less than 28%.

**Table 2.** Inhibition percentages exerted by compounds **1r** and **KIST101029** over 40-kinase panel including FMS kinase.

	% Inhibition^a–c^	
Kinase enzyme	1r	KIST101029
ABL1	15%	41%
ALK	0%	11%
Aurora A	−2%	21%
BTK	1%	7%
CaMKI	−23%	−1%
CDK2/cyclinE	−1%	−12%
CDK5/p25	−5%	8%
CHK1	−6%	−8%
c-MET	40%	−16%
c-SRC	−4%	10%
DMPK	−1%	−18%
EGFR (T790M)	4%	1%
EPHA1	−10%	9%
FGFR1	−3%	6%
FLT3 (D835Y)	42%	16%
FMS	81%	90%
FYN	14%	44%
GSK3β	−3%	4%
HIPK2	6%	9%
IGF-1R	4%	10%
IKKβ	1%	23%
IR	5%	2%
JNK1α1	−5%	−1%
KDR	1%	71%
LCK	−7%	33%
MEK1	−7%	12%
MLK1	−4%	−1%
mTOR	−2%	−10%
P70S6K	−8%	7%
PAK2	−9%	2%
PDK1	−7%	−1%
PIM1	−6%	10%
PLK1	7%	4%
RET	27%	57%
ROCK1	2%	3%
ROS/ROS1	−4%	19%
RSK1	−4%	8%
SAPK2a	−1%	25%
SYK	−5%	6%
TEC (activated)	10%	14%

^a^The compounds were tested at a single-dose concentration of 1 μM.

^b^100% Activity refers to enzyme activity in negative control (DMSO).

^c^% Inhibition was calculated by subtracting % activity from 100.Shade significe the most sensitive kinase.

#### Cell-based biological assays of compounds 1r and KIST101029

**Figure 2.**
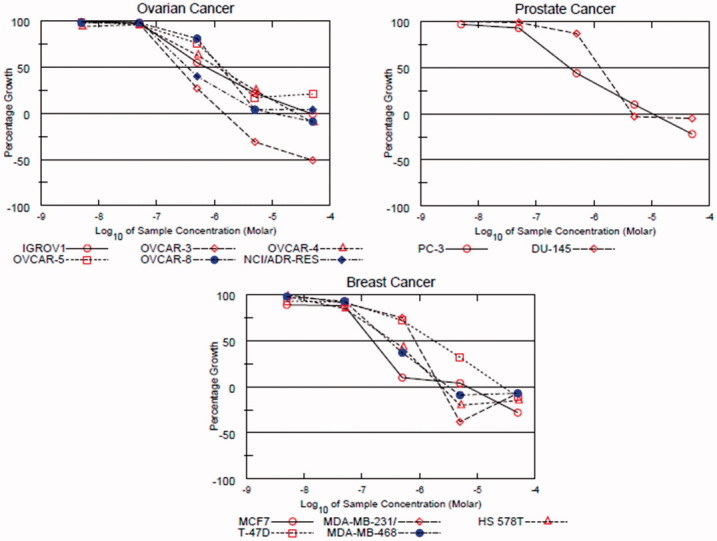
Dose-response curves of compound **1r** over the ovarian, prostate, and breast cancer subpanels.

Due to over-expression of FMS kinase in certain types of tumours such as ovarian^3^, prostate^4^, and breast^5–7^ cancers, we decided to test the antiproliferative activity of compound **1r** against a panel of six ovarian, two prostate, and five breast cancer cell lines. It was also tested against HS 27 fibroblasts in order to investigate the selectivity indexes. The results were compared with those of **KIST101029** as a reference standard. The IC_50_ values are presented in Table 3, and the dose-response curves of compound **1r** are shown in Figure 2.

**Table 3.** IC_50_ and TGI values in μM of compound **1r** and **KIST101029** over ovarian, prostate, and breast cancer cell lines.

	Cell line	5-dose results of Compound **1r**		5-dose results of **KIST101029**	
		IC_50_^a^	TGI^b^	IC_50_^a^	TGI^b^
Ovarian cancer	IGROV1	0.70	43.3	0.06	56.9
	OVCAR-3	0.24	1.47	0.02	0.05
	OVCAR-4	1.08	26.0	0.24	50.0
	OVCAR-5	1.38	>50	0.20	>100
	OVCAR-8	1.26	9.96	0.06	>100
	NCI/ADR-RES	0.34	>50	0.04	26.4
Prostate cancer	PC-3	0.38	10.40	0.06	>100
	DU-145	1.30	4.67	0.04	0.10
Breast cancer	MCF7	0.15	6.86	0.04	54.0
	MDA-MB-231/ATCC	0.84	2.32	0.16	3.42
	HS 578T	0.34	2.43	0.99	97.2
	T-47D	1.78	27.5	0.08	7.46
	MDA-MB-468	0.30	3.14	0.03	0.10
HS-27 Fibroblasts		5.72	-	0.30	-

^a^IC_50_ is the concentration producing 50% inhibition. The results are expressed as means of duplicate experiments.

^b^TGI is the concentration producing 100% inhibition.

At 10 µM concentration, compound **1r** was more active than **KIST101029** against four ovarian, two prostate, and two breast cancer cell lines (Supplementary file). **1r** was also more potent than **KIST101029** against HS 578 T breast cancer cell line. TGI values reflect the efficacy of the molecule. Compound **1r** was more efficacious than **KIST101029** against MCF7, MDA-MB-231/ATCC, and HS 578 T breast cancer cell lines. But in general, **KIST101029** was more potent than **1r** against most of the tested cell lines. The structural difference, benzamido in **KIST101029** compared with amino in **1r**, is responsible for this potency difference. The benzamido moiety is more hydrophobic and is expected to allow greater ability of the molecule to cross the cell membrane to inside the cell. So the exposure of the intracellular components to compound **KIST101029** is expected to be higher. Furthermore, the benzamido moiety may form additional interactions with the receptor site, which can contribute to stronger affinity and potency. However, the IC_50_ values of compound **1r** were in the range of 0.15–1.78 µM, which is an excellent range of potency. In addition, compound **1r** has another merit over **KIST101029**. Its IC_50_ value against HS 27 fibroblasts was higher, i.e. greater selectivity towards cancer cells than normal cells. Upon dividing its IC_50_ value against HS 27 fibroblasts by IC_50_ range over cancer cells, we find that the selectivity range of compound **1r** ranges from 3.21 to 38.13 times towards cancer cells than normal cells.

#### BMDM assay

FMS over-expression contributes to inflammatory disorders as explained in the Introduction section. So compounds **1r** and **KIST101029** were tested for their ability to inhibit CSF-1-induced BMDM growth. The IC_50_ values are summarised in Table 4. The more potent FMS kinase inhibitor **1r** exerted 2.32-fold superior potency than **KIST101029** in this assay. So, the compound **1r** can be a promising candidate for future development of anti-arthritic drugs.

**Table 4.** IC_50_ values of the tested compounds against bone marrow-derived macrophages (BMDM).

Compound No.	IC_50_ (nM)^a^
**1r**	84 ± 3
**KIST101029**	195 ± 6

^a^IC_50_ values are expressed as means of triplicate assay ± SEM.

Each compound was tested in a 10-dose testing mode, threefold serial dilution starting with 1 µM concentration.

## Conclusions

Our previous report on the potent and selective FMS kinase inhibitor, **KIST101029**, encouraged us to investigate the FMS kinase inhibitory effects of analogues of that lead compound. Eighteen compounds were tested, and this study led to discovery of compound **1r** as a more potent and selective FMS kinase inhibitor (3.2 times more potent than **KIST101029**). It showed strong potency against the tested ovarian, prostate, and breast cancer cell lines with IC_50_ values ranged from 0.15 to 1.78 µM. It selectivity index towards cancer cells than normal fibroblasts ranged from 3.21 to 38.13. In addition, compound **1r** showed potential anti-inflammatory effect against bone marrow-derived macrophages. This compound is a promising candidate for anticancer and anti-arthritic drug development. Further lead optimisation and biological investigations are required.

## Supplementary Material

Supplemental Material
