# Supplementary material for: Pyrrolo[3,2-c]pyridine derivatives with potential inhibitory effect against FMS kinase: in vitro biological studies
Source: J Enzyme Inhib Med Chem. 2018 Aug 2;33(1):1160–6. doi: 10.1080/14756366.2018.1491563 (PMC6084503; doi:10.1080/14756366.2018.1491563)
Supplement: Supplemental Material [file IENZ_A_1491563_SM0673.pdf]

Pyrrolo[3,2-*c*]pyridine derivatives with potential inhibitory effect against FMS kinase: *In vitro* biological studies

Mohammed I. El-Gamal [1,2,3] and Chang-Hyun Oh [4,5,*]

[1] Department of Medicinal Chemistry, College of Pharmacy, University of Sharjah, Sharjah 27272, United Arab Emirates.

[2] Sharjah Institute for Medical Research, University of Sharjah, Sharjah 27272, United Arab Emirates.

[3] Department of Medicinal Chemistry, Faculty of Pharmacy, University of Mansoura, Mansoura 35516, Egypt.

[4] Center for Biomaterials, Korea Institute of Science and Technology, PO Box 131, Cheongryang, Seoul 130-650, Republic of Korea.

[5] Department of Biomolecular Science, University of Science and Technology, 113 Gwahangno, Yuseong-gu, Daejeon 305-333, Republic of Korea.

[*] Corresponding author: Tel.: +82 2 958 5160; fax: +82 2 958 5308; E-mail address: choh@kist.re.kr; Address: Center for Biomaterials, Korea Institute of Science and Technology, PO Box 131, Cheongryang, Seoul 130-650, Republic of Korea [C.-H. Oh].

7-Hydroxy-1*H*-pyrrolo[2,3-*b*]pyridinium 3-chlorobenzoate (3)[22]

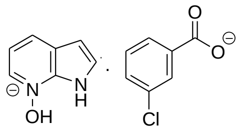

To a solution of 7-azaindole (**2**, 19.83 g, 167.9 mmol) in DME/heptane (1:2, 294 mL) was added 3-chloroperbenzoic acid (85 wt %, 46.2 g, 194.9 mmol) portionwise at 8 to 26 °C. Precipitation occurred after half of the 3-chloroperbenzoic acid was added. The slurry was stirred at room temperature for 2.5 h. The precipitate was filtered and washed with DME/heptane (1:2, 100 mL). The product was dried to yield an offwhite solid (43.58 g, 89.2%). mp: 141-143 °C (Lit. mp: 144.1-146 °C) [22].

4-Chloro-7-azaindole (4) [22]

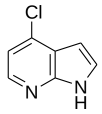

To compound **3** (43.4 g, 149.3 mmol) was added $POCl_3$ (170.4 g) at room temperature. The solution was heated to 55 °C, and then heating was removed. The temperature slowly went up to 74 °C in about 1 h without external heating. The mixture was further heated to 85-90 °C for 18 h. *Caution: Two exothermic events were observed, one at 50-60 °C and the other at 105-110 °C. To prevent a runaway reaction on scale, the solution was first heated to 55 °C and then heated slowly to 85-90 °C.* The solution was cooled to 50 °C, and $POCl_3$ was distilled off in vacuo. The residue was dissolved in acetonitrile (100 mL) and quenched by slow addition of water (100 mL) while keeping the temperature under 50 °C. The mixture was basified to pH 9 with 50% NaOH solution. The slurry was allowed to cool to room temperature, and the precipitates were filtered. The wet cake was reslurried with H_2O (200 mL), filtered, and dried to afford the product (18.15 g, 80%). mp: 176-177 °C (Lit. mp: 175.3-177 °C (Wang *et al.*, 2006)).

General procedure for preparation of compounds 5a,b

A mixture of compound **4** (230 mg, 2.0 mmol) and the appropriate nitroaniline derivative (10.0 mmol) was fused at 180 ºC for 2-5 h with stirring. The reaction mixture was cooled to room temperature and dissolved in ethanol (150 mL). The resulting suspension was filtered to

remove the insoluble material and the filtrate was evaporated under reduced pressure. The residue was purified by column chromatography (silica gel, ethyl acetate-methanol 10:1 v/v then switching to ethyl acetate-methanol 5:1 v/v) to obtain the desired purified products.

*1-(4-Nitrophenyl)-1H-pyrrolo[3,2-c]pyridin-4-amine hydrochloride (**5a**)*

Yield 18%; ^{1}H NMR (DMSO-d_6): δ 13.40 (brs, 1H), 8.42 (d, 2H, J = 8.9 Hz), 7.90 (d, 2H, J = 9.0 Hz), 7.80 (d, 1H, J = 5.9 Hz), 7.68 (d, 1H, J = 6.8 Hz), 7.34 (d, 1H, J = 6.3 Hz), 7.01 (d, 1H, J = 7.2 Hz); MS *m/z*: 255.95 (M^+ + 2), 254.65 (M^+).

*1-(3-Nitrophenyl)-1H-pyrrolo[3,2-c]pyridin-4-amine hydrochloride (**5b**)*

Yield 9.5%; ^{1}H NMR (CD_3OD): δ 8.33 (t, 1H, J = 2.0 Hz), 8.25 (dd, 1H, J = 1.1, 6.1 Hz), 7.94-7.91 (m, 1H), 7.81-7.76 (m, 1H), 7.63 (d, 1H, J = 2.2 Hz), 7.45 (d, 1H, J = 3.4 Hz), 6.90 (d, 1H, J = 3.4 Hz), 6.85 (d, 1H, J = 6.3 Hz); ^{13}C NMR (CD_3OD): δ 155.1, 150.4, 144.5, 140.3, 132.2, 131.0, 127.2, 122.6, 119.7, 115.0, 113.3, 104.5, 98.6; MS *m/z*: 255.95 (M^+ + 2), 254.65 (M^+).

General procedure for preparation of compounds 6a,b

To a stirred solution of compound **5a,b** (1.6 mmol) in acetonitrile (25 mL) at room temperature, diisopropylamine (0.7 mL, 4.0 mmol) was slowly added under nitrogen atmosphere. Benzoyl chloride (0.2 mL, 1.9 mmol) was slowly added and the reaction mixture was stirred at room temperature for 8 h. The reaction mixture was concentrated under reduced pressure, and water (20 mL) and CH_2Cl_2 (20 mL) were added to the residue. The organic layer was separated and the aqueous layer was extracted with CH_2Cl_2 (2 x 20 mL). The combined organic layer extracts were washed with brine, 1N HCl, and then aqueous $NaHCO_3$, dried over anhydrous $MgSO_4$, and filtered. The organic solvent was evaporated under reduced pressure and the residue was purified by column chromatography to give the desired products.

*N-(1-(4-Nitrophenyl)-1H-pyrrolo[3,2-c]pyridin-4-yl)benzamide (**6a**)*

It was purified by column chromatography (silica gel, hexane-ethyl acetate 3:1 v/v); yield 85%; ^{1}H NMR (DMSO-d_6): δ 8.49 (d, 2H, J = 5.0 Hz), 8.10 (d, 1H, J = 6.0 Hz), 7.88-7.82 (m, 6H), 7.63 (d, 1H, J = 6.0 Hz), 7.55-7.50 (m, 2H), 7.43-7.38 (m, 2H); MS *m/z*: 359.11 (M^+ + 1), 358.24 (M^+).

*N-(1-(3-Nitrophenyl)-1H-pyrrolo[3,2-c]pyridin-4-yl)benzamide (**6b**)*

Yield 15%; ^{1}H NMR (DMSO-d_6): δ 10.90 (brs, 1H), 8.41 (t, 1H, *J* = 2.1 Hz), 8.30 (d, 1H, *J* = 8.0 Hz), 8.16-8.09 (m, 4H), 7.93-7.82 (m, 2H), 7.62-7.52 (m, 4H), 6.74 (brs, 1H); MS *m/z*: 359.11 (M^+ + 1), 358.24 (M^+).

General procedure for preparation of compounds 7a,b

A mixture of compound **6a,b** (1.3 mmol) and Pd/C (10%) in anhydrous THF (20 mL) was stirred in hydrogen atmosphere at room temperature for 2 h. The reaction mixture was filtered through celite and the filtrate was concentrated under reduced pressure. The residue was purified by column chromatography to give the purified desired products.

*N-(1-(4-Aminophenyl)-1H-pyrrolo[3,2-c]pyridin-4-yl)benzamide (**7a**)*

It was purified by column chromatography (silica gel, hexane-ethyl acetate 1:2 v/v); yield 48%; ^{1}H NMR (DMSO-d_6): δ 10.78 (brs, 1H), 8.12-7.93 (m, 3H), 7.63-7.46 (m, 5H), 7.20 (d, 2H, *J* = 8.6 Hz), 6.75-6.62 (m, 3H), 5.39 (brs, 2H); ^{13}C NMR (DMSO-d_6): δ 164.4, 152.9, 145.0, 141.0, 133.4, 132.8, 132.5, 132.3, 129.0, 127.3, 124.7, 120.7, 117.5, 117.3, 104.7; MS *m/z*: 330.24 (M^+ + 2), 329.21 (M^+ + 1).

*N-(1-(3-Aminophenyl)-1H-pyrrolo[3,2-c]pyridin-4-yl)benzamide (**7b**)*

It was purified by column chromatography (silica gel, hexane-ethyl acetate 1:2 v/v) to give the purified desired product. Yield 32%; ^{1}H NMR (DMSO-d_6): δ 10.84 (brs, 1H), 8.11-8.09 (m, 3H), 7.62 (d, 2H, J = 3.2 Hz), 7.54 (d, 2H, J = 7.5 Hz), 7.42 (brs, 1H), 7.22 (t, 1H, J = 7.9 Hz), 6.77 (brs, 1H), 6.69-6.62 (m, 3H), 5.50 (brs, 2H); MS *m/z*: 330.24 (M^+ + 2), 329.21 (M^+ + 1).

General procedure for synthesis of compounds 1a-e

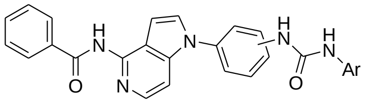

1a-e

To a solution of compound **7a,b** (0.06 mmol) in anhydrous THF (10 mL), the appropriate aryl isocyanate (0.06 mmol) was added. The reaction mixture was stirred under nitrogen atmosphere

at room temperature for 8 h. The solvent was evaporated under reduced pressure, and the residue was purified by column chromatography (silica gel, hexane-ethyl acetate 3:1 v/v then switching to hexane-ethyl acetate 1:1 v/v) to obtain the purified target product.

1-(4-(4-Benzamido-1H-pyrrolo[3,2-c]pyridin-1-yl)phenyl)-3-(3-(trifluoromethyl)phenyl)urea ***(1a)***

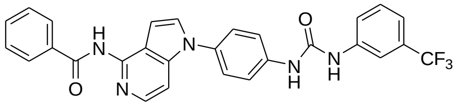

Yield 35%; mp: 158-160 °C; ^{1}H NMR (DMSO-d_6): δ 11.71 (brs, 1H), 10.83 (brs, 1H), 8.59-8.57 (m, 2H), 8.11-8.09 (m, 4H), 7.74 (d, 2H, J = 7.8 Hz), 7.66 (d, 2H, J = 3.2 Hz), 7.62-7.59 (m, 2H), 7.58-7.52 (m, 4H), 7.39 (d, 1H, J = 5.4 Hz), 6.66 (brs, 1H); MS *m/z*: 517.33 (M^+ + 2, 46.6%), 516.36 (M^+ + 1, 100%), 515.38 (M^+, 21%); elemental analysis: Calculated: C: 65.24%, H: 3.91%, N: 13.59%, Found: C: 65.08%, H: 3.99%, N: 13.54%.

1-(3-(4-(Benzamido)-1H-pyrrolo[3,2-c]pyridin-1-yl)phenyl)-3-(3-(trifluoromethyl)phenyl)urea ***(1b)***

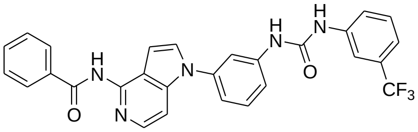

Yield 12.4%; mp: 147-148 °C; ^{1}H NMR (DMSO-d_6): δ 10.86 (brs, 1H), 10.31-10.24 (m, 2H), 8.25-8.09 (m, 4H), 8.04 (brs, 1H), 7.71 (d, 2H, J = 3.2 Hz), 7.66 (brs, 1H), 7.61-7.48 (m, 6H), 7.29 (d, 1H, J = 7.5 Hz), 7.22-7.20 (m, 1H), 6.68 (brs, 1H); ^{13}C NMR (DMSO-d_6): δ 164.8, 152.9, 141.3, 141.0, 139.8, 138.8, 132.8, 132.5, 132.3, 129.6, 129.1, 128.8, 128.7, 127.3, 127.0, 125.7, 125.2, 124.7, 123.6, 122.8, 121.9, 120.1, 119.5, 118.6, 117.2, 104.6; MS *m/z*: 517.33 (M^+ + 2), 516.36 (M^+ + 1), 515.38 (M^+); elemental analysis: Calculated: C: 65.24%, H: 3.91%, N: 13.59%, Found: C: 65.31%, H: 13.62%, N: 13.70%.

1-(4-(4-Benzamido-1H-pyrrolo[3,2-c]pyridin-1-yl)phenyl)-3-(4-chloro-3-(trifluoromethyl)phenyl)urea ***(1c)***

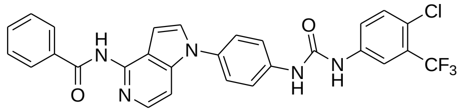

Yield 25%; mp: 204-206 °C; ^{1}H NMR (300 MHz, DMSO-*d*$_6$) δ 10.83 (brs, 1H), 9.37 (brs, 1H), 9.24 (brs, 1H), 8.15 (d, 1H, *J* = 2.0 Hz), 8.11-8.08 (m , 3H), 7.73-7.65 (m, 5H), 7.57-7.54 (m, 5H), 7.39 (d, 1H, *J* = 5.8 Hz), 6.65 (d, 1H, *J* = 3.2 Hz); ^{13}C NMR (75 MHz, DMSO-*d*$_6$) δ 164.8, 152.9, 141.0, 139.8, 139.0, 132.8, 132.5, 132.3, 129.7, 129.1, 128.8, 128.7, 127.3, 127.0, 125.7, 125.2, 124.7, 123.6, 122.8, 121.9, 120.1, 119.5, 117.3, 104.6; MS *m/z*: 552.22 (M + 2)$^+$, 551.18 (M^+ + 1), 550.14 (M^+); elemental analysis: Calculated: C: 61.15%, H: 3.48%, N: 12.73%, Found: C: 61.30%, H: 3.22%, N: 12.58%.

*1-(3-(4-Benzamido-1H-pyrrolo[3,2-c]pyridin-1-yl)phenyl)-3-(4-chloro-3-(trifluoromethyl)phenyl)urea (**1d**)*

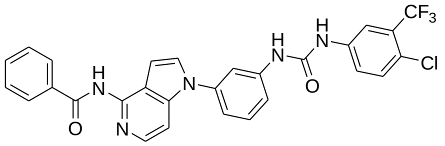

Yield 53%; mp: 143-145 °C; ^{1}H NMR (DMSO-*d*$_6$): δ 10.88 (brs, 1H), 9.85 (brs, 1H), 9.73 (brs, 1H), 8.15-8.09 (m, 3H), 7.90-7.87 (m, 1H) , 7.72-7.69 (m, 2H), 7.59 (d, 2H, *J* = 9.3 Hz), 7.55-7.48 (m, 4H), 7.25-7.19 (m, 1H), 6.87 (brs, 1H), 6.68 (d , 1H, *J* = 3.8 Hz); MS *m/z*: 552.22 (M^+ + 2), 551.18 (M^+ + 1), 550.14 (M^+); elemental analysis: Calculated: C: 61.15%, H: 3.48%, N: 12.73%, Found: C: 60.98%, H: 3.60%, N: 12.76%.

*1-(3,5-Bis(trifluoromethyl)phenyl)-3-(4-(4-benzamido-1H-pyrrolo[3,2-c]pyridine-1-yl)phenyl)urea (**1e**)*

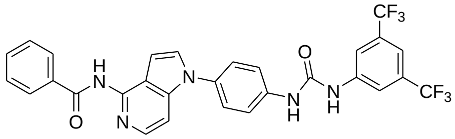

Yield 18%; mp: >280 °C; ^{1}H NMR (300 MHz, DMSO-*d*$_6$) δ 10.84 (brs, 1H), 9.50 (brs, 1H), 9.29 (brs, 1H), 8.17-8.03 (m, 4H), 7.73-7.67 (m, 4H) , 7.62-7.52 (m, 5H), 7.39 (d, 1H, *J* = 5.3 Hz), 6.86 (s, 1H), 6.65 (brs, 1H); ^{13}C NMR (300 MHz, DMSO-*d*$_6$) δ 168.1, 153.0, 141.1, 139.1, 132.2, 131.9, 129.0, 128.6, 127.8, 126.8, 125.3, 124.9, 124.3, 123.1, 122.3, 121.6, 120.1, 117.4,

104.9; MS *m/z*: 584.52 (M^+ + 1), 583.56 (M^+); elemental analysis: Calculated: C: 59.69%, H: 3.28%, N: 12.00%, Found: C: 59.80%, H: 3.20%, N: 12.05%.

General procedure for synthesis of compounds 1f-k

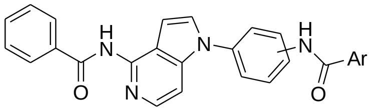

1f-k

A mixture of compound **7a,b** (0.06 mmol), the appropriate carboxylic acid derivative (0.12 mmol), HOBt (18.1 mg, 0.13 mmol), and EDCI (29.1 mg, 0.15 mmol) in dry DMF (1.0 mL) was cooled to 0 °C under nitrogen atmosphere. To the reaction mixture, triethylamine (0.002 mL, 0.015 mmol) was added at 0 °C. The mixture was then stirred at 80 °C for 12 h. The reaction mixture was cooled and then partitioned between water (5 mL) and ethyl acetate (5 mL) and the organic layer was separated. The aqueous layer was then extracted with ethyl acetate (3 x 3 mL), and the combined organic extracts were washed with brine and dried over anhydrous Na_2SO_4. After evaporation of the organic solvent, the residue was purified by column chromatography (silica gel, hexane-ethyl acetate 3:1 v/v then switching to hexane-ethyl acetate 1:1 v/v) to yield the purified target product.

*N-[4-(4-Benzoylamino-pyrrolo[3,2-c]pyridin-1-yl)phenyl]-3,4-Dichlorobenzamide (**1f**)*

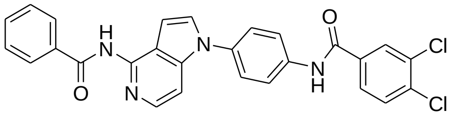

Yield: 15%; ^{1}H NMR (DMSO-d_6, 300 MHz) δ 11.23 (brs, 1H), 10.59 (brs, 1H), 8.67 (s, 1H), 8.27 (d, 1H, *J* = 1.7 Hz), 8.11-8.08 (m, 3H), 8.00-7.95 (m, 3H), 7.89-7.85 (m, 4H), 7.59-7.54 (m, 3H), 6.84 (d, 1H, *J* = 3.8 Hz); ESI-MS: 501.0 (M^+ + 1); elemental analysis: Calculated: C: 64.68%, H: 3.62%, N: 11.17%, Found: C: 64.47%, H: 3.51%, N: 11.32%.

*N-[3-(4-Benzamido-1H-pyrrolo[3,2-c]pyridin-1-yl)-phenyl]-3,4-dichlorobenzamide (**1g**)*

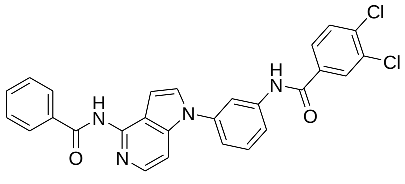

Yield: 15%; mp: 190-191 °C; ^{1}H NMR (DMSO-d_6): δ 10.86 (brs, 1H), 10.68 (brs, 1H), 8.26 (d, 1H, J = 2.0 Hz), 8.15-8.11 (m, 4H), 7.98 (dd, 1H, J = 2.0, 2.1 Hz), 7.88-7.85 (m, 2H), 7.59-7.52 (m, 3H), 7.45-7.39 (m, 3H), 6.76-6.63 (m, 2H); MS *m/z*: 501.50 (M^+ + 1), 500.46 (M^+); elemental analysis: Calculated: C: 64.68%, H: 3.62%, N: 11.17%, Found: C: 64.52%, H: 3.75%, N: 11.01%.

*N-[4-(4-Benzamido-1H-pyrrolo[3,2-c]pyridin-1-yl)-phenyl]-3-[(trifluoromethyl)]benzamide (**1h**)*

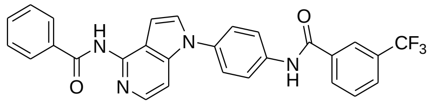

Yield: 16.4%; ^{1}H NMR (300 MHz, DMSO-d_6) δ 10.95-10.85 (m, 2H), 8.51 (s, 1H), 8.43-8.37 (m, 2H), 8.17-7.97 (m, 4H), 7.82-7.53 (m, 5H), 7.33-7.26 (m, 3H), 6.80 (d, 2H, J = 8.5 Hz); MS *m/z*: 501.50 (M^+ + 1), 500.46 (M^+); elemental analysis: Calculated: C: 67.20%, H: 3.83%, N: 11.19%, Found: C: 67.24%, H: 3.90%, N: 11.06%.

*N-[3-(4-Benzamido-1H-pyrrolo[3,2-c]pyridin-1-yl)-phenyl]-3-(trifluoromethyl)benzamide (**1i**)*

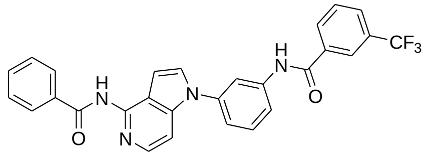

Yield 26%; mp: 142-144 °C; ^{1}H NMR (DMSO-d_6): δ 10.89 (brs, 1H), 10.77 (brs, 1H), 8.31 (d, 2H, J = 9.1 Hz), 8.18-8.09 (m, 2H), 8.02 (d, 1H, J = 7.6 Hz), 7.88-7.80 (m, 2H), 7.76 (d, 1H, J = 3.3 Hz), 7.68-7.58 (m, 4H), 7.59 (d, 2H, J = 8.6 Hz), 7.44-7.39 (m, 2H), 6.70-6.67 (m, 1H); MS *m/z*: 503.01 (M^+ + 2), 502.00 (M^+ + 1), 501.00 (M^+); elemental analysis: Calculated: C: 67.20%, H: 3.83%, N: 11.19%, Found: C: 67.36%, H: 3.74%, N: 11.00%.

*N-[4-(4-Benzamido-1H-pyrrolo[3,2-c]pyridin-1-yl)-phenyl]-4-morpholino-3-(trifluoromethyl)benzamide (**1j**)*

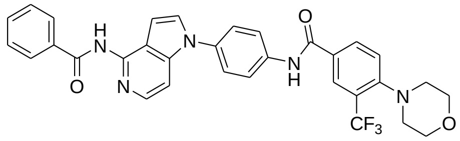

Yield: 11%; mp: 220-223 °C (dec.); ^{1}H NMR (300 MHz, DMSO-d_6) δ 10.92 (brs, 1H), 10.62 (brs, 1H), 8.43-8.28 (m, 2H), 8.11-8.05 (m, 2H), 8.01 (d, 2H, J = 8.8 Hz), 7.71 (d, 2H, J = 2.6 Hz), 7.68-7.52 (m, 4H), 7.50-7.44 (m, 2H), 7.29 (brs, 1H), 6.74-6.69 (m, 1H), 3.76 (t, 4H, J = 3.1 Hz), 2.96 (t, 4H, J = 3.3 Hz); ^{13}C NMR (75 MHz, DMSO-d_6) δ 164.5, 155.0, 141.0, 140.2, 134.3, 133.8, 133.5, 132.4, 130.9, 129.0, 128.8, 128.6, 127.5, 125.8, 124.8, 124.3, 123.1, 122.0, 119.8, 114.7, 104.8, 66.9, 53.6; MS m/z: 587.63 $(M + 2)^+$, 586.60 $(M^+ + 1)$, 585.56 (M^+); elemental analysis: Calculated: C: 65.63%, H: 4.48%, N: 11.96%, Found: C: 65.40%, H: 4.24%, N: 12.10%.

N-[4-(4-Benzamido-1H-pyrrolo[3,2-c]pyridin-1-yl)-phenyl]-3,5-bis(trifluoromethyl)benzamide ***(1k)***

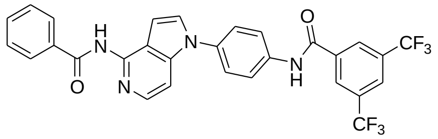

Yield 18%; ^{1}H NMR (300 MHz, DMSO-d_6) δ 10.84 (brs, 1H), 9.50 (brs, 1H), 8.17-8.03 (m, 4H), 7.73-7.67 (m, 4H) , 7.62-7.52 (m, 5H), 7.39 (d, 1H, J = 5.3 Hz), 6.86 (s, 1H), 6.65 (brs, 1H); ^{13}C NMR (300 MHz, DMSO-d_6) δ 168.2, 153.0, 141.1, 139.1, 132.2, 131.9, 129.0, 128.6, 127.8, 126.8, 125.3, 124.9, 124.3, 123.1, 122.3, 121.6, 120.1, 117.4, 104.9; MS m/z: 569.1 $(M^+ + 1)$, 568.1 (M^+); elemental analysis: Calculated: C: 61.27%, H: 3.19%, N: 9.86%, Found: C: 61.45%, H: 3.03%, N: 10.06%.

1-(4-Aminophenyl)-1*H*-pyrrolo[3,2-*c*]pyridin-4-amine (8a) and 1-(3-aminophenyl)-1*H*-pyrrolo[3,2-*c*]pyridin-4-amine (8b)

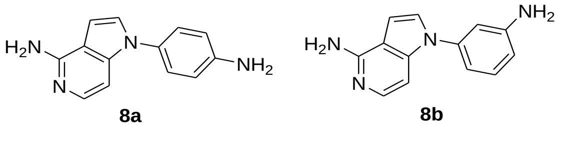

A mixture of **6a** or **6b** (0.47 g, 1.31 mmol) and $SnCl_2.H_2O$ (1.48 g, 6.55 mmol) in ethanol (20 mL) was heated under reflux for 2 h. The solvent was evaporated under reduced pressure, and the residue was partitioned between aqueous $NaHCO_3$ and ethyl acetate. The organic layer was separated, and the aqueous layer was extracted with ethyl acetate. The combined organic extracts were washed with brine and dried over anhydrous Na_2SO_4. After evaporation of the organic

solvent, the residue was purified by column chromatography (silica gel, ethyl acetate) to yield compound **8a** (0.12 g, 40.8%) or **8b** (0.15 g, 51%), respectively. MS *m/z*: 225.27 (M^+ + 1), 224.25 (M^+).

The diarylureas **1l,m** and the diarylamides **1n-r** were synthesized by the same method described for synthesis of compounds **1a-e** and **1f-k**, respectively.

*1-(4-(4-Amino-1H-pyrrolo[3,2-c]pyridin-1-yl)phenyl)-3-(4-chloro-3-(trifluoromethyl)phenyl)urea (**1l**)*

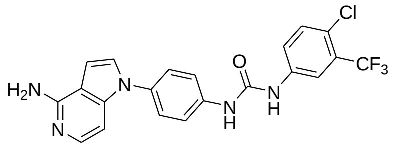

It was purified by column chromatography (silica gel, hexane-ethyl acetate 2:1 v/v then switching to hexane-ethyl acetate 1:3 v/v); yield: 27%; mp > 280 °C; ^{1}H NMR (300 MHz, CD_3OD) δ 8.06 (d, 1H, *J* = 2.4 Hz), 7.95 (d, 1H, *J* = 2.2 Hz), 7.72-7.64 (m, 4H), 7.59-7.51 (m, 3H), 7.44 (d, 2H, *J* = 3.6 Hz), 7.40-7.32 (m, 2H), 6.98 (d, 1H, *J* = 3.1 Hz), 6.87 (brd, 1H, *J* = 6.8 Hz); MS *m/z*: 447.90 $(M + 2)^+$, 446.86 (M^+ + 1), 445.82 (M^+); elemental analysis: Calculated: C: 56.57%, H: 3.39%, N: 15.71%, Found: C: 56.73%, H: 3.32%, N: 15.54%.

*1-(4-(4-Amino-1H-pyrrolo[3,2-c]pyridin-1-yl)phenyl)-3-(3,5-bis(trifluoromethyl)phenyl)urea (**1m**)*

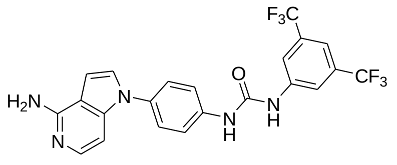

It was purified by column chromatography (silica gel, hexane-ethyl acetate 2:1 v/v then switching to ethyl acetate); yield: 8%; mp > 280 °C; ^{1}H NMR (300 MHz, DMSO-d_6) δ 8.56 (d, 1H, *J* = 6.6 Hz), 7.93-7.72 (m, 4H), 7.54-7.44 (m, 4H), 7.43-7.30 (m, 5H), 7.15 (d, 1H, *J* = 6.4 Hz); MS *m/z*: 480.10 (M^+ + 1), 479.10 (M^+); elemental analysis: Calculated: C: 55.12%, H: 3.15%, N: 14.61%, Found: C: 55.05%, H: 3.28%, N: 14.72%.

*N-(4-(4-Amino-1H-pyrrolo[3,2-c]pyridin-1-yl)phenyl)-3,4-dichlorobenzamide (**1n**)*

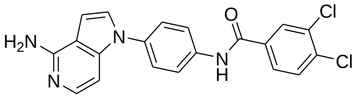

It was purified by column chromatography (silica gel, hexane-ethyl acetate 3:1 v/v then switching to hexane-ethyl acetate 1:1 v/v); yield: 12%; mp > 280 °C; ^{1}H NMR (300 MHz, DMSO-d_6) δ 8.07 (d, 1H, J = 2.3 Hz), 7.94 (d, 1H, J = 2.2 Hz), 7.70-7.63 (m, 4H), 7.59-7.50 (m, 3H), 7.44 (d, 2H, J = 3.7 Hz), 7.40-7.33 (m, 2H), 7.00 (d, 1H, J = 3.0 Hz); MS *m/z*: 398.0 (M + 2)$^+$, 397.0 (M$^+$ + 1), 396.0 (M$^+$); elemental analysis: Calculated: C: 60.47%, H: 3.55%, N: 14.10%, Found: C: 60.30%, H: 3.40%, N: 14.30%.

*N-(3-(4-Amino-1H-pyrrolo[3,2-c]pyridin-1-yl)phenyl)-3,4-dichlorobenzamide (**1o**)*

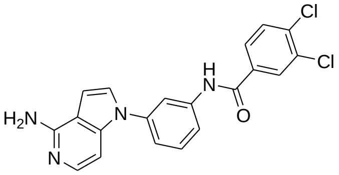

It was purified by column chromatography (silica gel, hexane-ethyl acetate 3:1 v/v then switching to hexane-ethyl acetate 1:1 v/v); yield: 11%; mp > 280 °C; ^{1}H NMR (300 MHz, DMSO-d_6) δ 8.20 (s, 1H), 8.06 (brd, 2H, J = 6.2 Hz), 7.85 (brd, 1H, J = 6.8 Hz), 7.74 (brd, 2H, J = 8.9 Hz), 7.69-7.61 (m, 2H), 7.51-7.40 (m, 4H), 7.31-7.18 (m, 2H); MS *m/z*: 398.14 (M + 2)$^+$, 397.13 (M$^+$ + 1), 396.12 (M$^+$); elemental analysis: Calculated: C: 60.47%, H: 3.55%, N: 14.10%, Found: C: 60.42%, H: 3.53%, N: 14.22%.

*N-(4-(4-Amino-1H-pyrrolo[3,2-c]pyridin-1-yl)phenyl)-4-morpholino-3-(trifluoromethyl)benzamide (**1p**)*

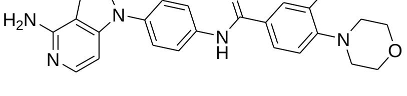

It was purified by column chromatography (silica gel, hexane-ethyl acetate 3:1 v/v then switching to ethyl acetate); yield: 37%; mp 253-256 °C (dec.); ^{1}H NMR (300 MHz, DMSO-d_6) δ 8.38 (d, 1H, J = 2.6 Hz), 8.29-8.26 (m, 2H), 8.02-7.99 (m, 2H), 7.75-7.51 (m, 5H), 7.20 (brd, 1H,

J = 8.0 Hz), 6.74 (brd, 1H, J = 8.2 Hz), 3.74-3.65 (m, 4H), 3.01-2.97 (m, 4H); MS *m/z*: 482.50 (M^+ + 1), 481.46 (M^+); elemental analysis: Calculated: C: 62.36%, H: 4.61%, N: 14.55%, Found: C: 62.18%, H: 4.47%, N: 14.71%.

*1-(4-(4-Amino-7H-pyrrolo[3,2-c]pyridin-7-yl)phenyl)-3-(3,5-bis(trifluoromethyl))benzamide (**1q**)*

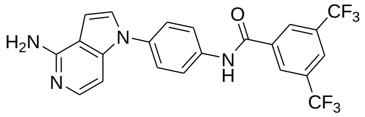

^{1}H NMR (DMSO-d_6, 300 MHz) δ 9.20 (s, 1H), 8.15 (s, 2H), 8.10 (s, 1H), 7.73-7.71 (m, 3H), 7.65 (brs, 1H), 7.62 (d, 2H, J = 8.9 Hz), 7.51 (d, 1H, J = 3.6 Hz), 7.13 (brs, 2H), 6.75 (d, 1H, J = 3.6 Hz); ESI-MS: 465.1 (M^+ + 1); elemental analysis: Calculated: C: 56.90%, H: 3.04%, N: 12.07%, Found: C: 56.75%, H: 2.94%, N: 12.23%.
